# Elevated nitrogen fertilization differentially affects jojoba wax phytochemicals, fatty acids and fatty alcohols

**DOI:** 10.3389/fpls.2024.1425733

**Published:** 2024-07-25

**Authors:** Zipora Tietel, Sarit Melamed, Izabella Galilov, Alon Ben-Gal, Arnon Dag, Uri Yermiyahu

**Affiliations:** ^1^ Department of Food Science, Gilat research Center, Agricultural Research Organization, Volcani Institute, Gilat, Israel; ^2^ The Robert H. Smith Faculty of Agriculture, Food and Environment, The Hebrew University of Jerusalem, Rehovot, Israel; ^3^ Gilat Research Center, Agricultural Research Organization, Volcani Institute, Rishon LeTsiyon, Israel

**Keywords:** *Simmondsia chinensis*, quality, health-related properties, wax esters, alternate bearing

## Abstract

Jojoba wax is gaining popularity among cosmetics consumers for its skin wound healing and rejuvenation bioactivities, attributed to collagen and hyaluronic acid synthesis. However, information regarding wax phytochemical composition and quality parameters, as well as effect of cultivation practices, and fertilization in particular, on wax quality is limited. The aim of the current work was to study the effect of nitrogen (N) availability to jojoba plants on wax phytochemical composition and beneficial skin-related contents. For this, wax quality from a six-year fertilization experiment with five N application levels was evaluated. The chemical parameters included antioxidant activity, free fatty acid, total tocopherol, total phytosterol and oxidative stability, as well as fatty acid and fatty alcohol profile. Our results reveal that the majority of wax quality traits were affected by N fertilization level, either positively or negatively. Interestingly, while fatty acids were unaffected, fatty alcohol composition was significantly altered by N level. Additionally, fruit load also largely affected wax quality, and, due to jojoba’s biennial alternate bearing cycles, harvest year significantly affected all measured parameters. Results shed light on the effects of N application on various biochemical constituents of jojoba wax, and imply that N availability should be considered part of the entire agricultural management plan to enhance wax quality. Some traits are also suggested as possible chemical quality parameters for jojoba wax.

## Introduction

Jojoba, *Simmondsia chinensis* L, is an ever-green Simmondsiaceae shrub, indigenous to southwestern North America and Central America, including Sonora, Mojavi, Colorado, Arizona and Baja California deserts. Jojoba is currently cultivated in various arid and semi-arid areas worldwide, including US, Mexico, Peru, Chile, Argentina, Australia, India, and Israel (Tietel et al., 2021a). Jojoba has an alternate-bearing yield pattern, i.e., it typically has a high-yield year (an “on-year”), followed by a low-yield year (an “off-year”). This natural behavior of jojoba plants might be suppressed using appropriate pruning methods (Lazare et al., 2021).

Jojoba seeds are extracted for their liquid wax, gaining interest as a desirable ingredient for the cosmetics industry, as well as an industrial lubricant. Jojoba wax is uniquely structured as an ester, comprising C12 to C24 mainly monounsaturated fatty acid (FA) chains, as well as C14 to C24 mainly monounsaturated fatty alcohols (FALs), esterified into C34-C50 ester molecules (Wisniak, 1977). This unusual chemical structure closely resembles that of the natural human skin sebum, which explains many of its skin-related beneficial effects. Traditionally, the wax was utilized by Native Americans for skin- and hair- related applications, e.g., for treating cuts, sores, bruises and burns, including sunburns and windburns (Dary, 2008). Our and others’ recent findings validate its ethnobotanical usages as a supreme skin care product, exhibiting anti-inflammatory (Habashy et al., 2005) and antioxidant properties (Abdel-Wahhab et al., 2016; Abou-Zeid et al., 2021), as a psoriasis (Nasr et al., 2017) and acne (Linder, 2008; Meier et al., 2012) treatment, its wound healing (Ranzato et al., 2011) and antiaging activities (Tietel et al., 2024), and its significant anti-herpes bioactivity (Tietel et al., 2021b). It has also been mentioned that Native Americans used the liquid internally to treat cancer and kidney disorders (Dary, 2008). Thus, further research regarding jojoba wax’s medicinal properties is warranted.

The question of jojoba seed and wax edibility is still open. Historic documentation show that Native American tribes ate the beans, roasted them make a coffee-like drink, and used them as an appetite suppressant when food was not available (Dary, 2008). The wax has been previously described as cooking oil (Gentry, 1958), with some works evaluating its potential as a non-calorie deep-frying oil (Saguy et al., 1996), and the Nestle Company performed some consumption trials, on which not much information is available (National Research Council, 1985; Baldwin, 1988). Nevertheless, some early studies involving animal feeding found growth retardation related to feeding inhibition and profound weight loss, which were attributed to jojoba cyanoglycoside simmondsin and its ferulate derivatives (Cokelaere et al., 1993, 1995). Some recent preliminary findings demonstrated jojoba’s traditional antidiabetic (Alsolami et al., 2023) and anti metabolic syndrome (Belhadj et al., 2020) properties, also supporting its anorexic effects (Belhadj, 2017; Belhadj et al., 2018). Nevertheless, jojoba wax is not currently used for food and is not generally recognized as safe (GRAS) by the US Drug Administration (USDA), although some current evidence might support its use as a food supplement, rather than a food ingredient.

Jojoba wax bioactivities are attributed to some of its phytochemical constituents, as its tocopherols (Lin et al., 2017), phytosterols (Lin et al., 2017; Wang and Wu, 2019), FAs and FALs (Verbiscar, 2004) have been acknowledged as contributing to skin-related bioactivity. Importantly, polyphenol and flavonoids, as well as simmondsin and derivatives, are present in various plant parts, but absent from the wax (Abdel-Mageed et al., 2014a). In addition, jojoba wax excels in its extreme oxidative stability, manifested by very long induction times compared to other plant oils (Torres et al., 2006). Although most produce cuticular wax, Jojoba is one of the very few instances of plants producing wax esters as a lipid reserve, in addition to *Limnanthes douglasii* (Isbell et al., 1996). Nonetheless, not much work has been done on characterizing jojoba wax quality parameters, and unlike other oil crops, e.g., olive oil, almost no common commercial parameters are available. The International Jojoba Expert Council (IJEC) has set various chemical standards, generally accepted by the manufacturers. However, the specific effects of these parameters, e.g., the detrimental effect of free fatty acids (FFA) on wax quality, or the contribution of specific FAs and FALs to quality and shelf life have not yet been determined, and are instead essentially based on the knowledge regarding olive oil.

Jojoba has been cultivated for its valuable wax since the 1960s, when it was domesticated as a plant-based substitute for banned sperm whale oil (Benzioni and Dunstone, 1986). Although some work has been performed on jojoba fertilization, previous studies focused mainly on growth and yield parameters, and failed to address the effect on wax quality (Nelson and Watson, 2001; Khattab et al., 2019; Hegab, 2021; Dag et al., 2023). As reported for other crops, nitrogen (N) fertilization majorly alters the chemical and phytochemical composition of the seed and oil, consequently affecting its quality (Erel et al., 2013; Verardo et al., 2013; Tietel et al., 2020). Currently, N fertilization rate common in commercial jojoba orchards in Israel is 250 kg/ha (Ben-Gal et al., 2024), and 150 kg/ha was found optimal with respect to fertilizer costs, crop productivity, and risk of environmental contamination with excess nitrate, as we previously showed (Dag et al., 2023). Hence, the aim of the current study was to investigate the effect of jojoba N availability on wax quality and beneficial parameters, focusing on its health-related contents, based on a six-year fertilization experiment. A secondary objective was to suggest chemical quality attributes for the wax, including establishing the contribution of FAs and FALs.

## Materials and methods

### Experimental set up

Details regarding the experimental design for the six-year jojoba fertilization experiment have been published previously (Dag et al., 2023). Briefly, the experiment was carried out in a 14-year-old commercial jojoba plantation (cv. Hatzerim) owned by Jojoba Israel near Beer Sheva (31°14′45.9″ N 34°43′18.2″ E). An experiment was designed to test fertilization treatments in a randomized-block statistical scheme, with five plots per block (a total of 25 plots), producing five plots per treatment (replications, n=5). Each plot was composed of three rows with nine plants in a row. Only the five middle plants in the middle row of each plot were measured. The rest of the plot was considered margins to reduce edge effects.

Separate irrigation and fertilization controllers and pumps were installed for the experiment at the beginning of 2016. Each fertilization treatment had its own fertilizer pump, water meter, and liquid-fertilizer storage container. Fertilizer solutions were prepared according to the treatment specifications and supplied by local fertilizer companies, Israel Chemical Ltd. and Deshen Gat.

Nitrogen fertilization was applied continuously and proportionally between March and November via subsurface drip irrigation system (fertigation). Nitrogen rates were relative to the expected seasonal irrigation volume, determined based on the 10-year daily average evapotranspiration from the Israel Meteorological Service data and commercially used crop factors. Treatments included five annual N application rates (50, 150, 250, 370, and 500 kg/ha). The ratio between ammonium and nitrate forms (N-NH_4_ and N-NO_3_, respectively) in the fertilization solution was about 1:1 in all treatments. P fertilization was based on P_2_O_5_, while K fertilizer was in the form of K_2_O (Dag et al., 2023). For all treatments, P and K were set at 100 and 300 kg/ha/year, respectively. Fertilization treatments were initiated on 22 June 2016, after 6 months of intentional N depletion with no fertilizer application.

On the current setup, 2017, 2019 and 2021 were “on” years, while 2016, 2018 and 2020 were “off” years (Dag et al., 2023).

### Seed sampling and wax extraction

Each year, a representative sample of 100 seeds was taken from the harvested yield of each plot after its cleaning. Jojoba wax was extracted from the seeds sample as previously reported (Tietel et al., 2024), by using a lab-scale cold press expeller in our lab (CA59, Komet, IBG, Moenchengladbach, Germany). The wax was extracted shortly after harvest. Lab-scale expeller was utilized for cold press (CA59, Komet, IBG, Moenchengladbach, Germany). Wax temperature was monitored during extraction, and did not exceed 40°C.

### Chemical analysis

#### FFA

FFA determination was carried out following analytical methods described in International Organization for Standardization (ISO) 660, as previously published (Tietel et al., 2019). Briefly, acid value was measured by titrating 1 gram of jojoba wax in isopropanol with KOH solution (0.05M in isopropanol), in the presence of phenolphthalein as an indicator, until a change of color was observed. KOH volume was recorded and FFA was expressed as mg KOH required to neutralize 1 g of wax (mg KOH/g wax).

#### DPPH

Antioxidant activity was measured using DPPH as previously described (Ananth et al., 2019). Briefly, one ml of DPPH (0.04 mM) solution was prepared in methanol and mixed with the wax (10 µl). After one hour of incubation at room temperature, the readings were measured spectrophotometrically at 517 nm using a 5-point trolox calibrating curve, and the results were expressed as TE/kg wax.

#### Oxidative stability

Oxidative stability of the wax was evaluated as previously reported (Tietel et al., 2019). Induction time was measured using a rancimat instrument (model 892, Metrohm, Switzerland), with 3 gr wax at 110°C, air flow rate of 10 l/hr.

#### Jojoba wax FA and FAL profiling

Jojoba wax FA and FAL profiling was performed based on our previously reported method (Tietel et al., 2021b). 50 µl wax samples were added to a 2 ml Eppendorf tube. One hundred and fifty µl of 3.2% sodium methoxide in methanol (w/v) were added and vortexed. Tubes were shaken in a thermoshaker (700 rpm) at 40°C for 30 min. Next, 100 µl of double-distilled water (DDW) was added, followed by an addition of 1000 µL hexane. The tubes were centrifuged for 2 min at 17,000 G. Eight hundred µl of the supernatant were then transferred to an injection vial supplemented with 200 µl of C17:0 (1 mg/ml, internal standard (IS)).

#### Chromatographic conditions

The samples, at 1 µl, were injected into an Agilent Technologies gas chromatograph (model 7890N) equipped with a mass spectrometer detector (model 5977). The carrier gas was helium, at a flow rate of 1 ml/min, on a DB-23 (60 m, 0.25 µm, 0.25 mm) column. The oven temperature was initially 175°C for 5 min, then increased to 240°C at 5°C/min, and held for 9.5 min. Inlet temperature was 250°C and split ratio 10:1. Fatty acids methyl esters (FAMEs) were identified by comparing retention times with those of standard compounds (FAMEs mix, Supelco, Sigma-Aldrich, Rehovot, Israel). The relative composition of the fatty acids in the waxes was determined as a percentage of total fatty acids (mass%). Fatty alcohols were annotated by using National Institute of Standards and Technology (NIST) 2014 library.

#### Total tocopherol content

Total tocopherol content was measured as previously described (Tietel et al., 2019). Briefly, wax samples (300 μl) were weighed into an Eppendorf tube, and 700 μl ethyl acetate were added, followed by 200 μl of FeCl3 solution (0.2% in ethanol (w/v)), and vortexed. Then, 200 μl of 2,2’-Dipyridinyl solution (0.2% in ethanol (w/v)) were added and mixed again. Tubes were then shaken in a thermoshaker (Thomas Scientific, Grant-bio, NJ, USA) at 25°C and 700 RPM for 20 min, covered with an aluminum foil. Two hundred μl were then added to a 96-well plate in three replicates, and samples read with a spectrophotometer (Thermo Fisher Scientific, MA, USA) at 520 nm. A six-point calibration curve based on 0-0.1 mg/ml concentrations of alpha tocopherol was used for calculations.

#### Total phytosterol content

Total phytosterol content was measured as previously described (Tietel et al., 2019). Briefly, wax samples (200 μl) were weighed into an Eppendorf tube, and 400 μl ethyl acetate were added and vortexed. In each of three replicates, 100 μl sample were added, followed by 100 μl Liebermann–Burchard (LB) reagent at -20 °C (made with 10 ml acetic anhydride and 1 ml H2SO4). Plates were covered with a designated plate sticker, covered with aluminum, foil and incubated at room temperature for 90 min, and then read at 675 nm. A six-point calibration curve based on 0-2 mg/ml concentrations of beta-sitosterol was used for calculations.

### Statistical analysis

Statistical analysis was performed using JMP16 (JMP Statistical Discovery, Cary, NC, USA). A multifactorial model using analysis of variance (ANOVA) was used, with N fertilization level, year (with “on”/”off” nesting) and plot as predictor variables, For trend determination, a five-level contrast analysis was performed (Rosenthal et al., 2000), and presented with its significance value, also when the trend was borderline significant (p<0.08), including its type (either linear (P_L_) or quadratic (P_Q_)), and the trendline itself. For correlations, linear regression was calculated, with R^2^ values above 0.3 considered as Fair (Akoglu, 2018).

## Results

### Phytochemical composition- quality parameters

**Figure 1:**
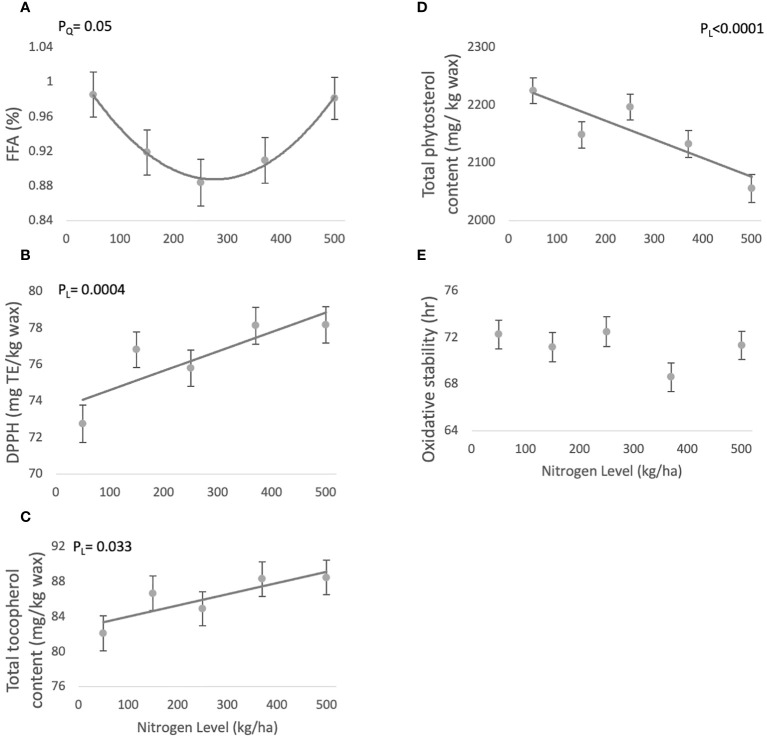
Effect of N fertilization on phytochemical composition of jojoba wax. Presented are means ± standard error (SE) of five plots (n=5). Lines are best fit quadratic **(A)** or linear regression **(B–D)**. **(A)** Free fatty acid (FFA) contents; **(B)** DPPH antioxidant activity; **(C)** Total tocopherol content; **(D)** Total phytosterol content; **(E)** Oxidative stability.

FFA levels response to N showed a borderline significant quadratic trend (P_Q_=0.0551) (**Table 1**), and varied in the range of 0.88 ± 0.02 and 0.98 ± 0.02 mg KOH/gr wax (**Figure 1A**).

**Table 1:** Statistical analysis from the ANOVA model.

	Treatment trend	Year	“On”/”Off” year
*Quality parameters*			
FFA (%)	P_Q_ =0.055	P<.0001	*
DPPH (mg TE/kg wax)	P_L_=0.0004	P<.0001	ns
Total Tocopherols (mg/kg wax)	P_L_=0.033	P<.0001	*
Total Phytosterols (mg/kg wax)	P_L_<.0001	P<.0001	ns
Oxidative stability (hr)	ns	P<.0001	*
*Fatty acids*			
C16:0 (%)	ns	P<.0001	*
C16:1 Δ^9^ (%)	ns	P<.0001	*
C18:1 Δ^9^ (%)	*(P_L_=0.057)*	P<.0001	*
C20:1 Δ^11^ (%)	*(P_Q_=0.061)*	P<.0001	ns
C22:1 Δ^13^ (%)	ns	P<.0001	*
C24:1 Δ^15^ (%)	*(P_L_=0.063)*	P<.0001	*
*Fatty alcohols*			
C18:1OH Δ^9^ %)	P_Q_ =0.039	P<.0001	*
C20:1OH Δ^11^ (%)	P_Q_ =0.009	P<.0001	ns
C22:1OH Δ^13^ (%)	P_Q_ =0.027	P<.0001	ns
C24:1OH Δ^15^ (%)	P_Q_ =0.032	P<.0001	*

ns, non significant.

* Statistically significant at p<0.05.

DPPH antioxidant activity values showed a significant linear increasing trend (P_L_=0.0004) with increasing N availability (**Table 1**), and varied between 72.75 ± 1.03 - 78.16 ± 0.99 mg TE/kg wax (**Figure 1B**).

Tocopherol levels increased linearly with increasing N (P_L_=0.0333) (**Table 1**), with levels in jojoba wax ranging between 82.07 ± 2.0- 86.67 ± 1.97 mg α-tocopherol/kg wax (**Figure 1C**).

Phytosterol levels significantly (P_L_<.0001) decreased with rising N levels (**Table 1**), with phytosterol content ranging between 2055.3 ± 24.2- 2224.6 ± 22.3 mg β-sitosterol/kg wax) (**Figure 1D**).

Oxidative stability did not present a particular trend in response to varying N levels (**Table 1**), and ranged 68.59 ± 1.24- 71.16 ± 1.24 hr (**Figure 1E**).

### Fatty acid and fatty alcohol profile

**Figure 2:**
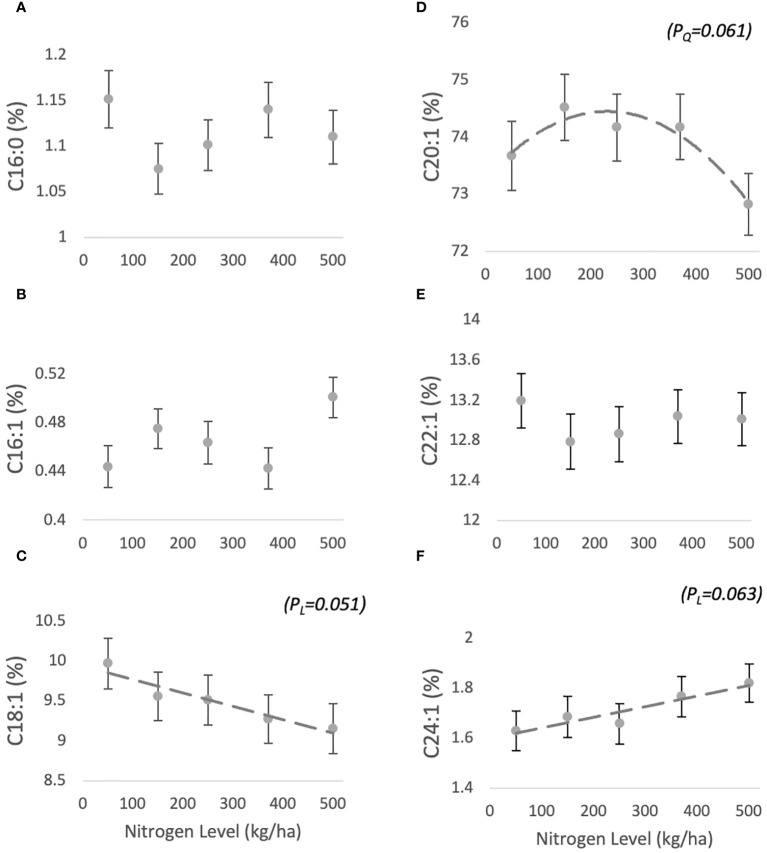
Effect of N fertilization on FA profile of jojoba wax. Presented are means ± standard error (SE) of five plots (n=5). Dashed lines are best fit (marginally significant) quadratic **(D)** or linear regression.

The fatty acid composition of jojoba wax comprises six main compounds: C16:0 (1.05 ± 0.02-1.17 ± 0.03%), C16:1 (0.47 ± 0.01-0.51 ± 0.01%), C18:1 (9.71 ± 0.31-10.5 ± 0.31%), C20:1 (72.18 ± 0.54- 73.92 ± 0.58%), C22:1 (12.96 ± 0.27- 13.35 ± 0.27%), C24:1 (1.57 ± 0.08-1.8 ± 0.07%) (**Figures 2A–F**, respectively). For C16:0 and C16:1 FA, no trend was found in response to N level (**Table 1**). C20:1 and C22:1 responses were characterized by quadratic trends (borderline (PQ=0.061) for the former and non-significant for the latter), with C20:1 peaking at 150 kg/ha and then decreasing, and C22:1 plunging at the same level and then increasing (**Figure 2**). C18:1 and C24:1 both responded with borderline linear trends, with C18:1 decreasing (P_L_=0.051) and C24:1 increasing (P_L_=0.063) with increasing N levels.

**Figure 3:**
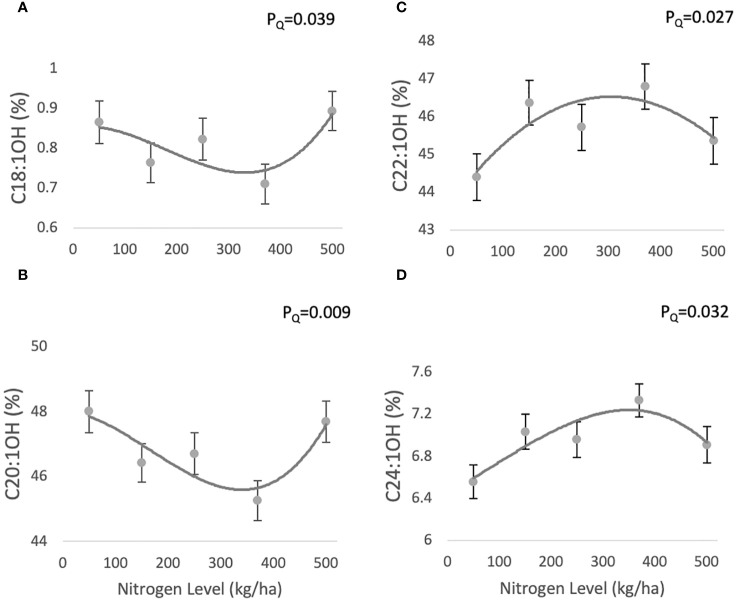
Effect of N fertilization on FAL profile of jojoba wax. Presented are means ± standard error (SE) of five plots (n=5). Lines are best fit quadratic regression.

The fatty alcohol composition of jojoba wax comprises four main compounds: C18:1OH (0.71 ± 0.04-0.8 ± 0.04%), C20:1OH (45.95 ± 0.61-48.07 ± 0.64%), C22:1OH (44.27 ± 0.61- 46.24 ± 0.6%), C24:1OH (6.29 ± 0.16-7.07 ± 0.15%) (**Figures 3A–D**, respectively). C18:1OH and C20:1OH responded according to a similar quadratic trend with increasing N (P_Q_=0.039 and P_Q_=0.009, respectively), decreasing at 370 kg/ha and then increasing, while C22:1OH and C24:1OH showed a complementary quadratic trend (P_Q_=0.027 and P_Q_=0.032, respectively), peaking at 370 kg/ha and then decreasing.

All measured parameters were significantly affected by year (**Table 1**), possibly due to differences in fruit load resulting from the biennial bearing “on”/”off” year effect, which was also significant for most parameters (**Table 1**). While the levels of some parameters, including total tocopherol content, total phytosterol content, oxidative stability, C18:1, C22:1, C18:1OH, and C22:1OH, were enhanced in “on” years the levels of others, including FFA, C16:0, C16:1, C24:1, and C24:1OH, or remained unaffected (antioxidant activity, C20:1, and C20:1OH), were higher in “off” years.

Correlations between measured parameters are presented in **Table 2**. FFA positively correlated to C20:1 and C24:1OH (low correlation), and negatively correlated to C18:1, C18:1OH, and C20:1OH. Antioxidant activity positively correlated with oxidative stability, with low negative correlation to C22:1, and C24:1. Total tocopherols positively correlated with oxidative stability, C18:1, C18:1OH and C20:1OH (low correlation with both latter), and negatively correlated with C20:1 and C24:1OH. Total phytosterol only negatively correlated with C24:1. Oxidative stability positively correlated with C18:1 and C20:1OH, and negatively with C20:1 (low correlation) and C22:1OH. All correlations were significant at P<0.0001.

**Table 2:** R^2^ values for parameter correlation.

	FFA	DPPH	Total tocopherols	Total phytosterols	Oxidative stability
FFA					
DPPH	ns				
Total tocopherols	ns	ns			
Total phytosterols	ns	ns	ns		
Oxidative stability	ns	0.302***	0.479***	ns	
C16:0	ns	ns	ns	ns	ns
C16:1	ns	ns	ns	ns	ns
C18:1	-0.316***	ns	0.687***	ns	0.366***
C20:1	0.299***	ns	-0.707***	ns	-0.257***
C22:1	ns	-0.193***	ns	ns	ns
C24:1	ns	-0.203***	ns	-0.403***	ns
C18:1OH	-0.362***	ns	0.236***	ns	ns
C20:1OH	-0.374***	ns	0.285***	ns	0.299***
C22:1OH	ns	ns	ns	ns	-0.399***
C24:1OH	0.284***	ns	-0.618***	ns	ns

ns, non significant.

***P<0.0001.

## Discussion

The available data regarding jojoba wax phytochemical composition is limited, and most of it dates back a few decades. As the informed reader might notice, while a large number of reviews are available for the wax (Sánchez et al., 2016; Al-Obaidi et al., 2017; Gad et al., 2021; Tietel et al., 2021a; El Gendy et al., 2023), concrete solid data is generally either scarce or entirely missing. Most available information regarding jojoba relates to the chemical rather than the phytochemical composition, including the wax, moisture, and ash content, alongside wax physical parameters. In the current work we were more interested in health-related bioactive compounds related to the beneficial effects of the wax. Other parameters, e.g., 100-seed weight, seed wax content and yield have been previously published for this experiment (Dag et al., 2023).

The acid value of cold-press jojoba wax has been reported as 0.36-1.0 mg KOH/g wax (Cappillino et al., 2003; Tobares et al., 2003; Gayol et al., 2009; Kaul et al., 2009; Shah et al., 2010; El-Boulifi et al., 2015; Bakeer et al., 2017; Agarwal et al., 2018), in line with the standard specifications of the IJEC of <1, although <2 is noted as a typical value (International Jojoba Export Council, 2024). Our findings align with these established ranges, suggesting similar cultivation conditions and genetic background. The effect of N fertilization on FFA levels was characterized by lower levels at moderate N exposure that escalated towards the extreme N treatments (0 and 500 kg/ha), potentially indicating a plant stress response triggered by extreme deficit or excess N (Ahmad et al., 2023b). In oil-bearing plants, stress response often induces lipase enzymatic breakdown of triglyceride (TAGs) backbone to release FAs, which serve as stress signaling molecules and antioxidants, to enable consequent biosynthetic processes and encounter high levels of reactive oxygen species (ROS) (Welte and Gould, 2017; He and Ding, 2020; Zienkiewicz and Zienkiewicz, 2020). In turn, this breakdown leads to enhanced FFA levels, as we and others have reported for olive oil (Tietel et al., 2019; Zipori et al., 2023). In jojoba, the same lipase reaction seems to take place, albeit involving jojoba esters rather than TAGs, to release FFAs (Kawiński et al., 2021). In olive oil, FFA levels increased with increasing fruit N, although in a linear manner, unlike our quadratic tendency (Erel et al., 2013).

In oilseeds and oil crops, high FFA values are regarded as important chemical quality indicators, inversely related to oil quality. Our findings thus also have implications concerning the commercial value of jojoba wax, underscoring the significance of N availability, not only in influencing the quality of fresh wax quality, but also potentially impacting its shelf life, as reported for other oils (Frega et al., 1999), although the specific effects of FFA on jojoba wax storability remain to be elucidated.

Peer-reviewed reports for jojoba wax *in-vitro* antioxidant activity are unavailable, although seed, leaf, and residue, as well as non-peer-reviewed and *in-vivo* results exist (Elnimiri and Nimir, 2011; Wagdy and Taha, 2012; Al-Qizwini et al., 2014a; Abdel-Mageed et al., 2014b; Al-Qizwini et al., 2014b; Abdel-Wahhab et al., 2016; Manoharan et al., 2016; Kara, 2017; Belhadj et al., 2018; Siahaan et al., 2020). In addition, the DPPH antioxidant activity of jojoba wax has only been reported as % inhibition, while we are reporting it here in mg TE/kg wax for the first time. Using Trolox as a standard for calibration curve to quantify DPPH activity enables a more accurate, absolute, repeatable, and comparable evaluation, which is why it was chosen over other antioxidant activity measuring methods. In addition, the DPPH free radical is compatible with apolar environments, which also makes it suitable for the wax (Prevc et al., 2013).

Information regarding the effect of N fertilization on antioxidant activity is minimal. In sesame seeds, antioxidant activity decreased with increasing N fertilization from 0-160 kg/ha (Elhanafi et al., 2019), in rice it increased between 0- 21 and then decreased until 420 kg/ha (Ma et al., 2023), and in Jerusalem artichoke increased between 10-120 kg/ha (Amarowicz et al., 2020). However, increased antioxidant activity has been reported for other crops under stress conditions (Tietel et al., 2019; Abdalla et al., 2022; Ahmad et al., 2023a). Inducing antioxidant capacity under stress-causing conditions enables plants to better cope with the enhanced oxidative status and encounter higher ROS levels (Dumanović et al., 2021). The observed increase in wax antioxidant activity with increasing N thus assumedly reflects jojoba plant response to elevated oxidative conditions induced by N stress, and is hence concomitant with the increase in FFA between 250-500 kg/ha. These results imply that the antioxidant response to N is crop- and range-dependent, and possibly reflects the specific plant’s stress response.

Unlike most of the other parameters evaluated in this work, antioxidant activity does not reflect the levels of a single compound, but rather encompasses the wax overall antioxidant capacity, emerging from the various compounds displaying antioxidant ability, whether known or unknown. Importantly, changes in wax antioxidant activity possess implications for wax quality, as antioxidant capacity underlies antioxidant bioactivity *in-vivo*, which has been reported for the wax (Abdel-Wahhab et al., 2016). Unequivocally, wax antioxidant activity holds significance for wax chemical quality and stability as well, also implied by their correlation (**Table 2**). Antioxidants mitigate the negative effects of FFA, peroxides and other factors by quenching free radicals present or forming in the oil due to oxidation processes (e.g., during storage or resulting from exposure to unfavorable conditions such as heat, light or oxygen) (Farhoosh and Hoseini-Yazdi, 2014), thus limiting oxidation and maintaining wax quality.

Jojoba wax total tocopherol content was reported in only two studies, as 63.3 mg/kg wax (Tobares et al., 2003) or 417 mg/kg (El-Mallah and El-Shami, 2009), both in line with our data. An increase in tocopherol content with N fertilization has been reported for other oil crops as well (Fernández-Escobar et al., 2006; Egesel et al., 2008; Verardo et al., 2013; Hussain et al., 2014; Carrera and Seguin, 2016; Aires et al., 2018). Tocopherols have been shown to play a significant role in mitigating and alleviating stress, improving adaptation and increasing tolerance by regulating plant physiology and immunity (Stahl et al., 2019; Ali et al., 2022), e.g., by maintaining the fluidity and integrity of photosynthetic membranes. The recorded increase in tocopherols with increasing N is thus suggested as another aspect of plant response to stress (Sadiq et al., 2019; Ali et al., 2020). This increase is also in accordance with the enhanced antioxidant activity, as expected with tocopherols being the most common antioxidants and specifically the most prevalent apolar antioxidants in the wax, to the best of our knowledge. Nevertheless, while research in other oil crops pointed out an over-fertilization effect, resulting in tocopherol decrease with high N levels, this was not observed in the current work, despite exposure to high N levels. Jojoba plants have been postulated to possess high tolerance to various abiotic stress conditions, e.g. heat, salinity and drought (Zheng et al., 2022; Al-Dossary et al., 2024), which might also explain its ability to also withstand high N levels without over-fertilization effects. While the results of this study suggest that jojoba plants subjected to extreme N treatments exhibited a stress response, they nonetheless maintained proper functioning and vitality, possibly indicating a lower susceptibility to the detrimental effects of such severe conditions.

As antioxidants, tocopherols play a twofold role; these compounds protect oil against oxidation, primarily as powerful antioxidants attributed to the antioxidant stability and antioxidant activity (Caddeo et al., 2018), and thus are an important oil quality parameter; furthermore, they are significant in protecting skin against cell membrane lipid oxidation, thus resisting inflammation (Zainal et al., 2022). Due to their important roles both in wax quality and skin-related benefits, the increase in tocopherols with increased exposure to N indicates improved wax quality, with enhanced bioactive properties. Although tocopherol response to N shared the same trend as antioxidant activity, no correlation was observed between the two parameters. This can be due to the fact that tocopherol antioxidant activity is not well measured by the DPPH radical, as it has been reported that the capacity of vitamin E to scavenge DPPH radical is low (Xie and Schaich, 2014).

Total phytosterol content in jojoba wax has been reported as 3148- 3977 mg/kg wax (Busson-Breysse et al., 1994; Shah et al., 2010; Al-Qizwini et al., 2014b), in accordance with our recorded values. Inconsistency exists within previous reports regarding the impact on oilseed phytosterols, which are either unaffected (Verardo et al., 2013), decrease (as in the current work) (Gul et al., 2007), or increase as a function of increased N fertilization (Kirkhus et al., 2013; Arbonés et al., 2020). However, no mechanism has been suggested for any of these responses, neither in oilseeds nor in cereals (Pavlík et al., 2010; Ma et al., 2023). The variation can possibly be attributed to the type and rate of fertilizer, soil N, the crop under question and its N requirements, as well as other environmental or growth conditions. Due to their important skin health related bioactivity (Tietel et al., 2024), a decrease in phytosterol levels is expected to adversely affect the beneficial pro-skin health properties of jojoba wax.

Although not usually regarded as classic antioxidants, phytosterols do exhibit some antioxidant-related bioactivity in plants (Liu et al., 2021), also promoting membrane integrity, possibly owing to their wide structural diversity (Rogowska and Szakiel, 2020). However, they do not seem to contribute to wax stability or antioxidant activity. Our data suggest that phytosterols might be consumed or converted, or that the decrease might be a result of either unfavorable stress (Ferrer et al., 2017), or diversion of assimilate flux towards other pathways (Erel et al., 2013). The observed decrease is presumed to impair wax quality, as these compounds are considered a wax quality attribute, suggested as skin beneficial and protective by maintaining membrane stability and integrity (Moalla Rekik et al., 2016), thus attenuating skin psoriatic inflammation (Chang et al., 2023), and promoting wound-healing and anti-aging (Wang and Wu, 2019).

Jojoba wax oxidative stability was reported as 41.3-60 hr (induction time) at 10 l/hr and 110°C (Kampf et al., 1986; Shah et al., 2010; El-Boulifi et al., 2015), similar to our data. The limited literature on the effect of N availability on oil oxidative stability in other oil crops shows a mixed trend, with either no effect (Marcelo et al., 2010; Kachel et al., 2019), a decrease and then an increase (Verardo et al., 2013), an increase (Amato et al., 2015), or a decrease (Pascual et al., 2019), while our data did not present a specific pattern. Unlike other attributes, oil stability is not a standalone parameter, but rather a measurement that integrates many other oxidation-related properties of the oil and its composition, since it depends on specific levels of antioxidants and prooxidants (e.g. metal ions), and FFA, in addition to lipid and FA composition (Shahidi and Zhong, 2010). As such, stability is highly affected by environmental and cultivation conditions, alongside oil production and storage conditions. These effects, if contradicting, might result in the absence of a clear trend, as could be the case here. Oxidative stability has immense commercial significance, as it binds all these factors into a linear influence on oil storability and shelf-life. This parameter represents a clear case where chemical quality resonates the health-related quality, as the constituents that chemically stabilize the wax, imparting longer induction time, are the same as those contributing to the beneficial health effects, as was also demonstrated in olive (Bernardini and Visioli, 2017).

The magnitude of differences reported between N treatments is sometimes minimal, as often observed in such works. However, these are generally unrelated to total wax yield, since as we previously reported, in most years no significant yield differences were recorded between treatments, and when present, differences were inconsistent (Dag et al., 2023). In addition, N fertilization rate did not influence the seed wax content.

The positive and negative correlations identified in this work between trends of the various measured parameters might assist in shedding light on the role of FA and FAL in jojoba wax quality. While in olive oil, high C18:1 level is favorable from an oxidative stability perspective, alongside low levels of PUFAs, which enhance oxidation, in jojoba these roles are yet to be established. Evidently, C18:1, C18:1OH and C20:1OH seem to be represent positive attributes, correlating with tocopherol and oxidative stability, while negatively correlating with FFA levels. Respectively, C20:1, C22:1, C24:1, C22:1OH, and C24:1OH show the opposite trend, negatively correlating with antioxidant activity, total tocopherol content, total phytosterol content, and/or oxidative stability. As expected, both antioxidant activity and total tocopherols positively correlate with antioxidant activity, all contributing to wax oxidative stability. These novel findings establish the roles of FAs and FALs in regard to wax quality, generally hinting that short chains imply better wax quality, while longer chains may impair jojoba wax positive attributes, although further research is required to describe these functions.

Jojoba wax fatty acid and alcohol profiles have been reported by us and others (Miwa, 1971; Clarke and Yermanos, 1980; Busson-Breysse et al., 1994; El-Mallah and El-Shami, 2009; Gayol et al., 2009; Shah et al., 2010; Awad et al., 2022; Tietel et al., 2024), and are generally quite consistent among publications, also in agreement with our current findings. The different reports present only slight variations, which could arise from differences in genetic background or cultivation conditions, although some studies reported significantly altered profiles (Arya et al., 2016; Agarwal et al., 2018). Curiously, none of the recorded FA responded with significant trends as a function of increasing N. Nevertheless, they all paired into three complementary trends: C16:0 and C16:1 FA; C20:1 and C22:1; and C18:1 and C24:1. The FALs also were separated into two groups of complementary trends: C18:1OH and C24:1OH; and C20:1OH and C22:1OH.

These complementary trends hint that these compounds are mutually increasing/decreasing, compensating for each other’s alternations, e.g., the increase in C24:1OH between 50 and 370 kg/ha is compensated by the concomitant decrease in C18:1OH. Surprisingly, FAs and FALs with the same chain length did not follow a similar trend, however they did pair to the same chain length compound in both cases: i.e., C18-C24 and C20-C22. Such balance might imply a common biosynthetic pathway.

To date, jojoba wax ester biosynthetic pathway has been elucidated, including enzyme substrate specificity (Miklaszewska and Banaś, 2016), showing that 18:1-CoAs from plastid FA synthase are elongated by FA elongase (FAE) to C20, C22 and C24 CoAs, which in turn are recued into the corresponding alcohols by fatty acyl reductase (FAR). FAs and FALs are then combined into C34-C50 wax esters by wax synthase (WS). Intriguingly, C42, the most abundant ester comprising 50-74% of the wax, is formed by esterification of either C20 together with C22 chains, or C18 and C24 chains (Wisniak, 1994). To maintain these proportions, C20 and C22, as well as C18 and C24, which are esterified together, need to present complementary trends (Miwa, 1971; El-Mallah and El-Shami, 2009), confirming our observations. Interest in that pathway is emerging, aiming at desirable plant-based esters as coatings and lubricants (Rowland and Domergue, 2012; Demski et al., 2022), encouraging jojoba wax ester biosynthesis in other transgenic organisms, e.g., *S. cerevisiae* (Wenning et al., 2019).

In olive, enhanced fruit N concentrations led to increased oil C18:2 and C18:3 levels, a decrease in C18:1 (Erel et al., 2013), and increased desaturation levels. Similarly results were reported for walnut (Verardo et al., 2013). We postulate that the results in jojoba are only partially comparable to those in other oil crops, due to the unique ester composition (rather than TAGs), which creates a different chemical environment, in addition to the different chain lengths and degrees of saturation compared to most other oils. However, it can be assumed that the quadratic trends observed for FALs reflect an optimized N response curve, demonstrating an optimum N range for each compound. The fact that while FALs are altered by N levels FAs remain unmodified might hint that the N levels influence FAR, whereas other enzymes of the ester biosynthetic pathway, e.g., FA synthase and elongase, are unaffected. Alternations in FALs probably indicate changes in substrate specificity of jojoba FARs, favoring longer fatty acyl CoAs chains (C22 and C24) in higher N levels (up to 370 kg/ha). Nevertheless, further research is still required to elucidate the exclusive impact of N levels on jojoba FAR.

The year effect was significant for all measured parameters, which is often observed in field experiments, probably due to year-to-year variation resulting from varying environmental conditions. Another aspect of the year was the bearing cycle (“on”/”off” year) effect, caused by jojoba alternate fruit bearing. Olive is another oil crop with biennial bearing cycles, for which previous studies have reported a similar negative effect of fruit load on FFA, showing higher FFA value in “off” years (Bustan et al., 2014; Pascual et al., 2019), as well as higher C16:0, C16:1, and C18:2 levels, alongside higher polyphenol content, C18:0 and C18:1 levels in “on” years (Ben-Gal et al., 2011). This corroborates our data for all common parameters (including tocopherols, equivalent to olive oil polyphenols as the main antioxidants). The higher levels of antioxidants and C18:1 can explain the higher oxidative stability and lower FFA, although while in olive oil the lower FFA was also attributed to FA acid profile, mainly C18:1 antioxidant activity, the contribution of FA and FAL profile to FFA and oxidative status in jojoba wax is yet to be determined. In addition, in olive, variation in FFA levels was also attributed to high fruit moisture content, increasing fruit sensitivity to mechanical harm. However, as jojoba wax is extracted from the seed rather than fruit pulp, such causation is less reasonable, and further research is required in order to elucidate the role of bearing cycle in FFA level determination.

In light of these findings, we can evaluate the bearing cycle effect, integrating the effects on the various quality parameters measured in this work. While “on” year wax had higher levels of five positive attributes (total tocopherol content, total phytosterol content, oxidative stability, and C18:1) and higher levels of two negative attributes (C22:1 and C22:1OH), “off” year wax had high levels of one positive attribute (C18:1OH) in addition to three negative attributes (FFA, C24:1, and C24:1OH). We can thus speculate that the wax quality of the high bearing “on” years is superior to that of low seed bearing “off” years, given that the contribution of these parameters is equal. Practically, these data imply that “on” year wax is higher quality due to lower FFA and higher oxidative stability as well as higher levels of quality attributes, e.g., tocopherols and phytosterols.

In conclusion, the current work demonstrates for the first time that N fertilization poses significant implications for jojoba wax quality. Moderate N fertilization, up to 150-250 kg/ha, improves wax chemical quality, including FFA, tocopherol content, and antioxidant activity, as well as the levels of high quality- related FAs and FALs, while phytosterol content decreases linearly and the oxidative stability is not significantly affected. Levels beyond these do not impart further improved quality, although, unlike for other crops, jojoba wax is generally not adversely affected by over-fertilization, plausibly as part of its remarkable overall tolerance to stress. Notably, the levels found to be optimal for wax quality were also favorable in regard to yield, N use efficiency (NUE), and preventing N transport into groundwater, as we previously reported (Dag et al., 2023). Our novel observations also reveal the role of specific FAs and FALs as wax positive attributes, although further work is required to determine their specific contributions to wax quality. We established jojoba wax chemical quality parameters as a first step in establishing jojoba commercial quality parameters similar to those accepted in olive oil.

## Data availability statement

The raw data supporting the conclusions of this article will be made available by the authors, without undue reservation. The JMP 16 parameters used in this work are available upon request.

## Author contributions

ZT: Conceptualization, Data curation, Formal analysis, Funding acquisition, Methodology, Supervision, Visualization, Writing – original draft, Writing – review & editing. SM: Data curation, Investigation, Methodology, Project administration, Validation, Writing – review & editing. IG: Investigation, Methodology, Writing – review & editing. ABG: Conceptualization, Formal analysis, Writing – review & editing. AD: Conceptualization, Funding acquisition, Writing – review & editing. UY: Conceptualization, Funding acquisition, Writing – review & editing.

## Supplementary material

Supplementary Figure 1 A GC-MS chromatogram of jojoba wax FAs and FALs.
