# Supplementary figures and images for: Elevated nitrogen fertilization differentially affects jojoba wax phytochemicals, fatty acids and fatty alcohols
Source: Front Plant Sci. 2024 Jul 25;15:1425733. doi: 10.3389/fpls.2024.1425733 (PMC11310937; doi:10.3389/fpls.2024.1425733)

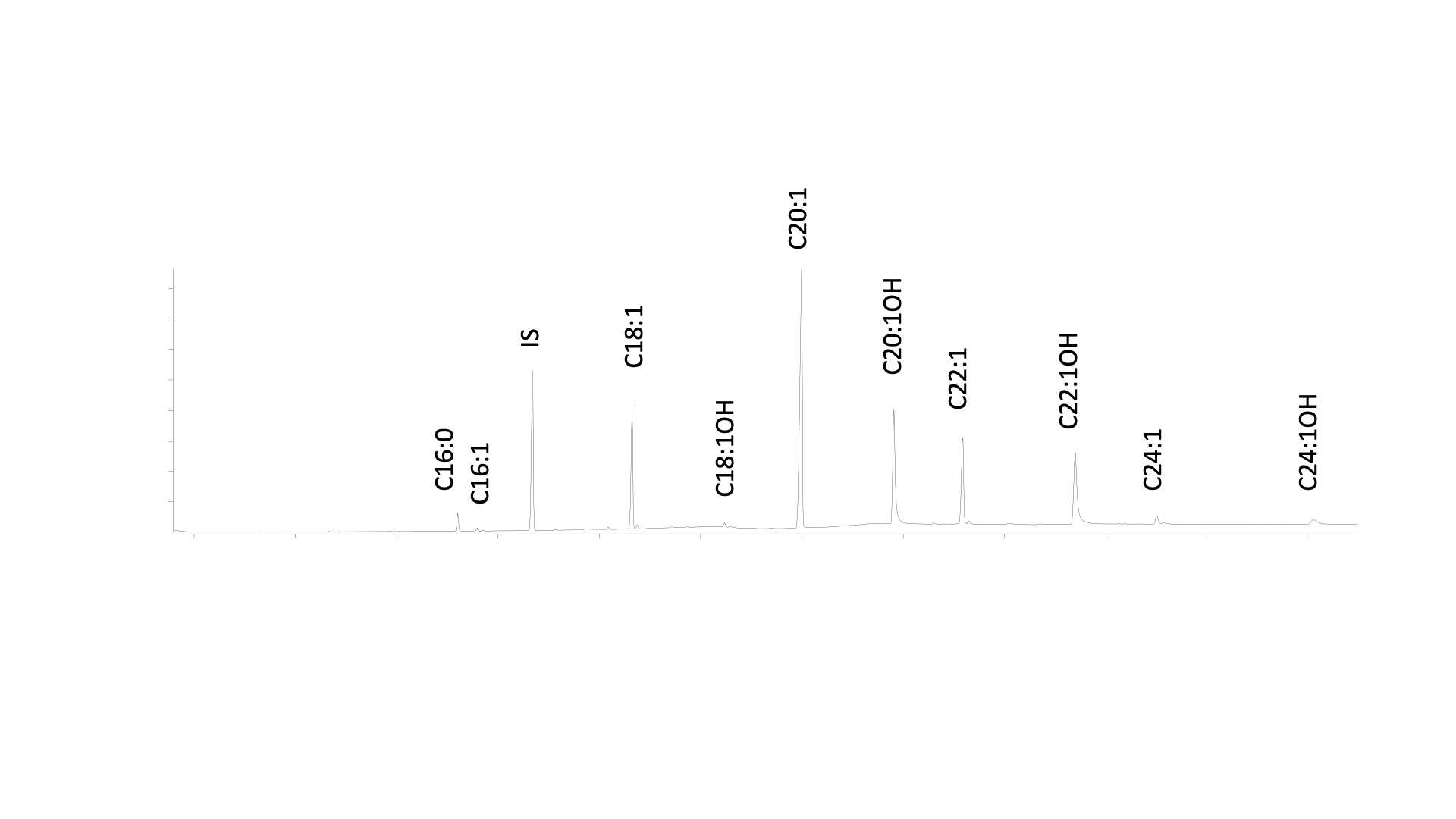

Supplement: Supplementary Figure 1 — A GC-MS chromatogram of jojoba wax FAs and FALs. [file Image_1.jpeg]
